# Stress Relaxation Analysis Facilitates a Quantitative Approach towards Antimicrobial Penetration into Biofilms

**DOI:** 10.1371/journal.pone.0063750

**Published:** 2013-05-27

**Authors:** Yan He, Brandon W. Peterson, Marije A. Jongsma, Yijin Ren, Prashant K. Sharma, Henk J. Busscher, Henny C. van der Mei

**Affiliations:** 1 Department of Biomedical Engineering, W.J. Kolff Institute, University Medical Center Groningen and University of Groningen, Groningen, The Netherlands; 2 Department of Orthodontics, University Medical Center Groningen, University of Groningen, Groningen, The Netherlands; Tel Aviv University, Israel

## Abstract

Biofilm-related infections can develop everywhere in the human body and are rarely cleared by the host immune system. Moreover, biofilms are often tolerant to antimicrobials, due to a combination of inherent properties of bacteria in their adhering, biofilm mode of growth and poor physical penetration of antimicrobials through biofilms. Current understanding of biofilm recalcitrance toward antimicrobial penetration is based on qualitative descriptions of biofilms. Here we hypothesize that stress relaxation of biofilms will relate with antimicrobial penetration. Stress relaxation analysis of single-species oral biofilms grown *in vitro* identified a fast, intermediate and slow response to an induced deformation, corresponding with outflow of water and extracellular polymeric substances, and bacterial re-arrangement, respectively. Penetration of chlorhexidine into these biofilms increased with increasing relative importance of the slow and decreasing importance of the fast relaxation element. Involvement of slow relaxation elements suggests that biofilm structures allowing extensive bacterial re-arrangement after deformation are more open, allowing better antimicrobial penetration. Involvement of fast relaxation elements suggests that water dilutes the antimicrobial upon penetration to an ineffective concentration in deeper layers of the biofilm. Next, we collected biofilms formed in intra-oral collection devices bonded to the buccal surfaces of the maxillary first molars of human volunteers. *Ex situ* chlorhexidine penetration into two weeks old *in vivo* formed biofilms followed a similar dependence on the importance of the fast and slow relaxation elements as observed for *in vitro* formed biofilms. This study demonstrates that biofilm properties can be derived that quantitatively explain antimicrobial penetration into a biofilm.

## Introduction

In the 17^th^ century the Dutch fabric merchant Antonie van Leeuwenhoek started to construct his own microscopes in order to be able to better examine the quality of the fabrics he bought and sold. He examined more than just his fabrics and after utilizing one of his own microscopes in 1684 to look at the accumulation of matter on his teeth, he remarked in a report to the Royal Society of London: "The number of these animalcules in the scurf of a man's teeth are so many that I believe they exceed the number of men in a kingdom". This was not enough however, to satisfy the curiosity of the fabric merchant, who would become one of the most famous microbiologists of all times, and he furthermore discovered “that the vinegar with which I washt my Teeth, kill’d only those Animals which were on the outside of the scurf, but did not pass thro the whole substance of it”.

Translated to one of the important topics in modern microbiology, Van Leeuwenhoek was referring to the biofilm mode of growth of bacteria adhering on a surface [1], embedding themselves in a matrix of extracellular polymeric substances (EPS) [2] that not only offers physical protection against antimicrobial penetration but can also yield bacterial properties that are different from their planktonic counterparts. Bacteria in their adhering, biofilm mode of growth can become inherently resistant to antimicrobials through mutation [3], formation of antibiotic degrading enzymes [4], endogenous oxidative stress [5], phenotypic changes [6], and low metabolic activities [7]. Despite extensive studies over many centuries, prevention of biofilm formation remains a prime challenge in many industrial and biomedical applications. In industrial applications, biofilms inflict major damage when formed on processing equipment or in pipes used to transport resources [8]. In the biomedical field, biofilm-related infections can develop everywhere in the human body from head (oral biofilms [9]) to toe (infected diabetic foot ulcers [10]). Biofilm-related infections are rarely cleared by the host immune system and especially infections that arise after implantation of biomaterial implants (e.g. prosthetic hips and knees) or devices (e.g. pace makers) are known to be persistent and difficult to treat, since the antimicrobial tolerance of bacteria in their biofilm mode of growth extends to many antibiotics used in modern medicine [11]. Moreover, dental caries and periodontal diseases, the most wide-spread infectious diseases in the world, are due to biofilms that Van Leeuwenhoek tried to eliminate by using vinegar as an antimicrobial mouthrinse [12].

Although the microscopes used nowadays are more sophisticated than the ones Van Leeuwenhoek employed, our understanding of the recalcitrance of biofilms toward antimicrobial penetration is still based on qualitative description of biofilms [13], using expressions as “water channels”, “mushroom structures”, “whiskers” and “streamers” [14], [15]. This raises the question whether quantifiable properties of biofilms exist that would relate with antimicrobial penetration into a biofilm. As for polymeric materials, structural and compositional properties of biofilms, should be reflected in their viscoelastic properties. Visco-elastic properties of oral biofilms depend on the degree of compaction during formation, the absence or presence of flow during growth, their architecture and microbial composition [16], [17]. The viscoelastic properties of oral biofilms can be determined by evaluating their relaxation after deformation during external loading. Stress relaxation during external loading is a time-dependent process and can be separated into a number of responses, each with a characteristic time-constant [18]. Although Maxwell analysis of stress-relaxation to derive the characteristic time-constants of the various relaxation processes that occur in a biofilm under external loading has been done before [19], results have been regarded mainly from a mathematical perspective and the details of the relaxation-structure-composition relation in biofilms and the physical processes associated with the different time-constants, are mostly neglected. Stress relaxation may involve a number of processes, like the outflow of water and EPS from the biofilm and re-arrangement of the bacteria in the biofilm [20]. Since penetration of an antimicrobial into a biofilm depends on diffusion [21] and therewith on its structural and compositional features, like the presence of water-filled channels in the biofilm or EPS-containing spaces, we here hypothesize that the penetration of an antimicrobial into a biofilm may relate with stress relaxation and its underlying processes.

The aim of this study is to gain evidence in support of this hypothesis. To this end, single-species biofilms of two oral bacterial strains, *Streptococcus oralis* and *Actinomyces naeslundii* were grown in a parallel plate flow chamber (PPFC) [22] and in a constant depth film fermenter (CDFF) [23]. Subsequently, we measured their visco-elastic properties using a low load compression tester, as well as the penetration of chlorhexidine into the biofilms. Following Van Leeuwenhoek, we chose to collect support for our hypothesis based on oral biofilms, because the human oral cavity is highly accessible and also allows for sampling of *in vivo* formed biofilm. Therefore, in order to not only gain *in vitro* evidence in support of our hypothesis, an intra-oral biofilm collection device was developed to grow oral biofilms *in situ*, in absence of mechanical perturbation. *In vivo* formed biofilms in the devices worn by human volunteers were examined *ex situ* with respect to their visco-elastic properties and chlorhexidine penetration and results and conclusions compared with those obtained for *in vitro* formed oral biofilms. Chlorhexidine is known to be the most effective oral antimicrobial to date [24] and surprisingly, despite its extensive use, inherent bacterial resistance against chlorhexidine has hardly or never been reported as compared to antibiotic resistance of many bacterial pathogens. This makes chlorhexidine an ideal antimicrobial to separate a possible inherent tolerance of biofilm bacteria for the antimicrobial from the physical protection offered by the biofilm mode of growth and study its penetration through a biofilm.

## Results

Biofilms of coccal-shaped *S. oralis* J22 and rod-shaped *A. naeslundii* T14V-J1 grown in the PPFC reached a thickness of 131±15 µm and 109±26 µm, respectively (Table 1). The biofilm thickness in the CDFF for *S. oralis* J22 was 119±6 µm and 125±9 µm for *A. naeslundii* T14V-J1. There were no significant differences (p>0.05, Student *t*-test) in thickness between biofilms grown under flow and in the CDFF. Also differences in biofilms thickness across strains were not statistically significant (p>0.05, Student *t*-test).

**Table 1:** The thickness, penetration ratio, total stress relaxation and the relative importance of the three Maxwell elements of *in vivo* and *in vitro* formed oral biofilms.^1.^

	Thickness(µm)	Penetrationratio	10% deformation (%)				20% deformation (%)				50% deformation (%)			
			Relaxation	E_1_	E_2_	E_3_	Relaxation	E_1_	E_2_	E_3_	Relaxation	E_1_	E_2_	E_3_
*In vivo*	121±86^a^	0.20±0.1*	60±14*	21±16*	27±13	52±16*	58±15*	24±14*	15±13	61±17*	65±11	43±16^a^	14±9	43±16
*In vitro*average	120±52	0.46±0.1	82±14	44±20	28±15	28±19	79±15	54±22	18±10	28±16	72±19	54±26	13±6	34±24
PPFCaverage	109–131^b^	0.33^b^–0.56	64–97^b^	17–60^b^	35^b^–40	4^b^–43	57–92^b^	25–73^b^	11^b^–26	16^b^–49	43–76^b^	18–65^b^	11^b^–15	24^b^–68
CDFFaverage	119^b^–125	0.39–0.48^b^	83^b^–83	47^b^–49	10^b^–25	26–43^b^	80^b^–84	56–60^b^	10^b^–27	17–30^b^	74^b^–90	59^b^–75	10^b^–14	11–31^b^

1 *In vivo* data refer to averages ± SD obtained in five volunteers, while *in vitro* data are averages over all single-species biofilms formed in the PPFC and CDFF by coccal and rod-shaped organisms. In addition, *in vitro* data are averaged as formed in the PPFC and CDFF by coccal and rod-shaped organisms.

* indicates p<0.05.

a indicates the comparison was carried out by Mann-Whitney Rank Sum test.

b indicates data for *S. oralis* J22.

The penetration of chlorhexidine in biofilms grown in the PPFC was significantly different (p<0.05, Mann-Whitney Rank Sum test) for *S. oralis* J22 and *A. naeslundii* T14V-J1, and the penetration ratio amounted 0.33±0.09 and 0.56±0.08, respectively (see also Table 1 and Fig. S1). On the other hand, there were no significant strain-dependent differences in penetration of chlorhexidine into biofilms grown in the CDFF, showing penetration ratios of 0.48±0.04 and 0.39±0.06 in biofilms of *S. oralis* J22 and *A. naeslundii* T14V-J1, respectively (p>0.05, Mann-Whitney Rank Sum test). Interestingly, whereas biofilms offered a clear physical protection against chlorhexidine, bacteria dispersed from biofilms grown either in the PPFC or in the CDFF were highly susceptible to chlorhexidine (Fig. S2), confirming that the absence of bacterial killing in the deeper layers of the biofilms are not due to changes in inherent properties of the bacteria in their biofilm mode of growth, but solely to difficulties encountered by the antimicrobial in penetrating to the deeper layers. Note that a similar conclusion has been drawn for three days old *in vivo* grown oral biofilms, after dispersal and exposure to chlorhexidine [25].

**Figure 1:**
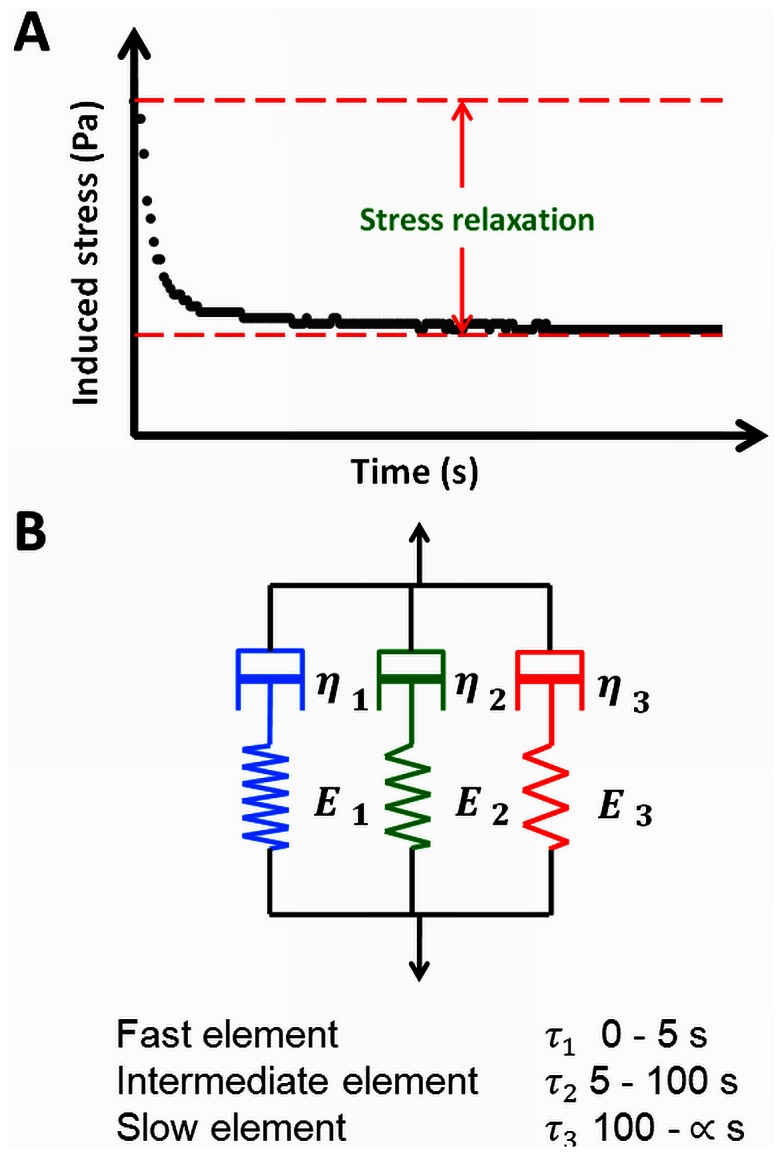
Measurement and Maxwell model of the viscoelasticity of biofilms. (A) Stress *versus* time diagram for relaxation of a compressed biofilm. (B) Schematic of a three element Maxwell model: E_i_ represent the spring constants and τ_i_ the relaxation time constants, which are equal to η_i_/E_i_.

**Figure 2:**
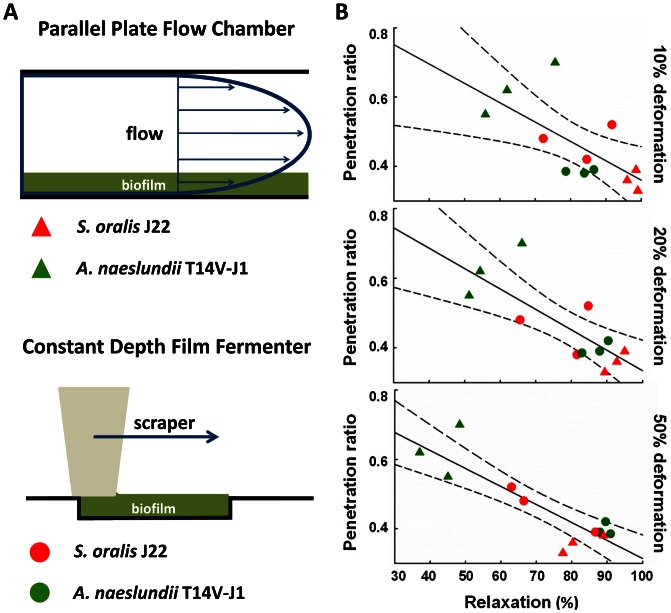
Penetration of chlorhexidine and stress relaxation of differently grown biofilms *in vitro*. (A) The schematics of parallel plate flow chamber and constant depth film fermenter. (B) Penetration ratio of chlorhexidine as a function of relaxation of different biofilms for 10%, 20% and 50% induced deformation. Dashed lines indicate 95% confidence intervals.

Total stress relaxation (Fig. 1A) of biofilms grown in the PPFC were different for both strains and *S. oralis* J22 biofilms showed significantly (p<0.05, Mann-Whitney Rank Sum test) more stress relaxation than biofilms of *A. naeslundii* T14V-J1, especially after 10% and 20% induced deformation (Table 1). There were no significant differences (p>0.05, Mann-Whitney Rank Sum test) in stress relaxation between biofilms of the coccal and rod-shaped organisms when grown in the CDFF. Interestingly, the penetration ratio of chlorhexidine decreased with increasing stress relaxation of the biofilms, regardless of the induced deformation (Fig. 2).

**Figure 3:**
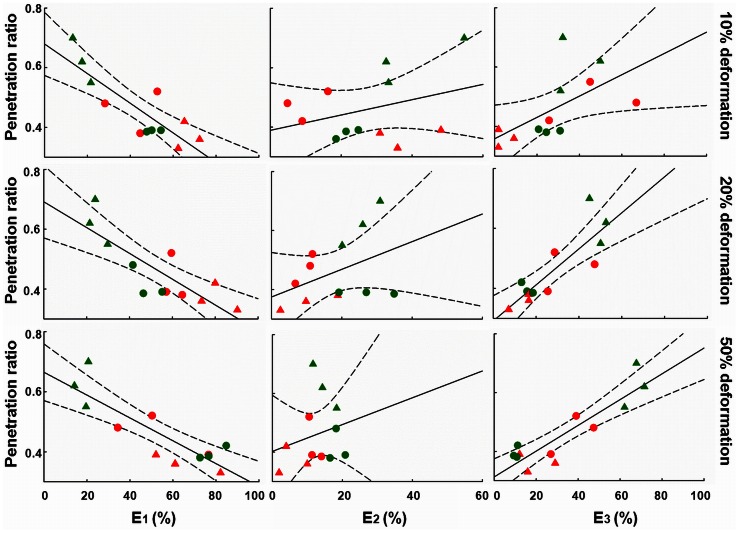
Chlorhexidine penetration and Maxwell analyses of *in vitro* grown biofilms. Penetration ratio as a function of the relative importance of the three Maxwell elements E_1_, E_2_ and E_3_, denoting the fast, intermediate and slow relaxation components, respectively for different biofilms after 10%, 20% and 50% induced deformation. All data points refer to single experiments, while symbols are explained in Fig. 2. Dashed lines represent 95% confidence intervals.

Total stress relaxation was subsequently resolved in a fast, intermediate and slow component (Fig. 1B). Since bacteria in a biofilm constitute the heaviest masses, their re-arrangement upon an induced deformation will be slow, and we associate the relative importance of the slow Maxwell element with bacterial re-arrangement in a biofilm. On the other hand, water has the smallest viscosity in a biofilm, and therefore the fast Maxwell element is associated with the flow of water through a biofilm, which leaves an association between the behavior of EPS with the intermediate Maxwell element. Analysis of the stress relaxation according to a three element Maxwell model revealed that penetration increased with increasing relative importance of the slow relaxation component and decreasing importance of the fast component (Fig. 3). This confirms the existence of a relaxation-structure-composition relation that may facilitate a quantitative approach towards antimicrobial penetration in biofilms.

In order to confirm that a relaxation-structure-composition relation facilitates understanding of antimicrobial penetration also for *in vivo* grown biofilms, we first developed an intra-oral biofilm collection device (Fig. S3). The average thickness of the oral biofilms formed *in vivo* over a time period of two weeks was 121±86 µm, comparable to the thickness of *in vitro* biofilms (p>0.05, Mann-Whitney Rank Sum test), as can be seen in Table 1.

**Figure 4:**
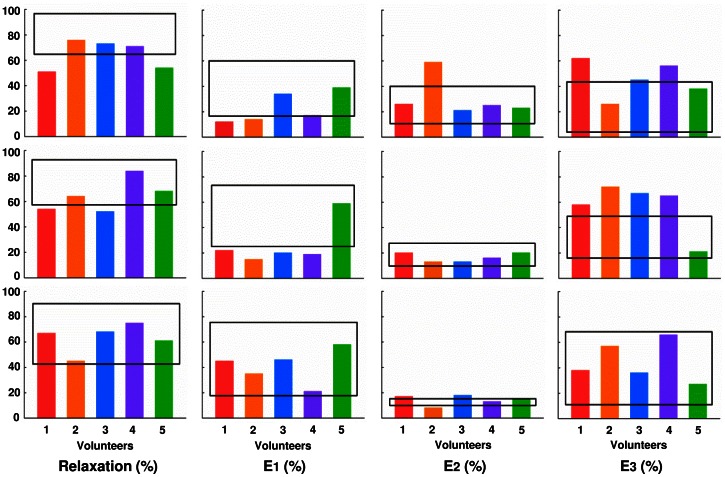
Stress relaxation properties of intra-orally grown oral biofilms. The *in vivo* biofilms were obtained in five volunteers as indicated by different colors in comparison with the average relaxation properties of different single-species biofilms formed in a PPFC and CDFF, falling within the black rectangles.

Total stress relaxation of *in vivo* biofilms upon 10% and 20% deformation were more comparable to the stress relaxation observed for *in vitro* biofilms grown in the PPFC than in the CDFF, as averaged over both bacterial strains (Table 1). On the other hand, upon inducing a deformation of 50%, stress relaxation of *in vivo* biofilms became more comparable to the one of *in vitro* biofilms grown in the CDFF. On average, *in vitro* biofilms showed higher total stress relaxation than *in vivo* formed biofilms, although this difference was only significant (p<0.05, Student *t*-test) for 10% and 20% induced deformations (Fig. 4).

*In vivo* formed biofilms furthermore distinguished themselves significantly from *in vitro* averages by a smaller importance of the fast component (E_1_) and larger importance of the slow component (E_3_) (p<0.05, Student *t*-test; Table 1) for induced deformations of 10% and 20%. At 50% induced deformation however, differences in the importance of the different relaxation parameters had disappeared (see also Fig. 4). The importance of the intermediate component (E_2_) was relatively similar across the different biofilms (Table 1).

**Figure 5:**
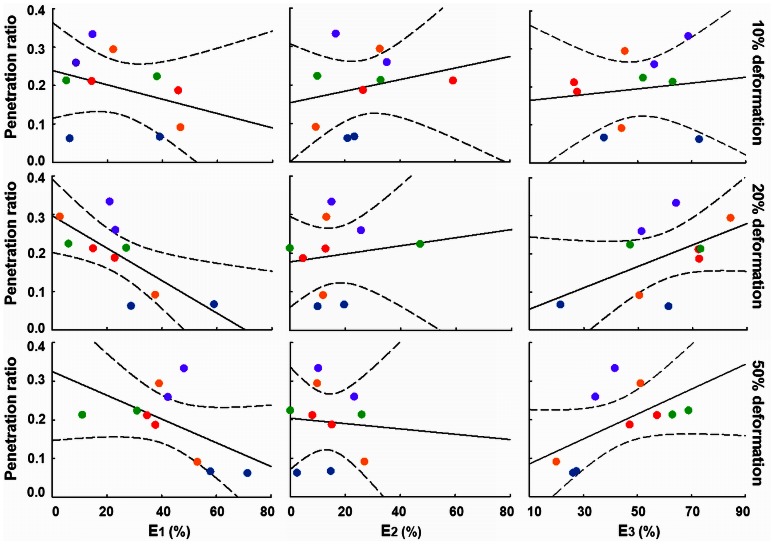
Chlorhexidine penetration and Maxwell analyses of intra-orally grown biofilms. Penetration ratio of chlorhexidine as a function of the relative importance of the fast, intermediate and slow Maxwell elements E_1_, E_2_ and E_3_ for *in vivo* biofilms formed in different volunteers after 10%, 20% and 50% induced deformation. All data points refer to single experiments in one volunteer. Different volunteers are indicated by the same color codes as used in Fig. 4. Dashed lines represent 95% confidence intervals.

The chlorhexidine penetration ratio for *in vivo* formed biofilms was smaller than the average penetration into *in vitro* biofilms (p<0.05, Student *t*-test; Table 1). Similarly as observed for *in vitro* biofilms, penetration decreased with increasing importance of the fast (E_1_) component and increased with the importance of the slow component (E_3_) (Fig. 5). No relation was observed with the importance of the intermediate component (E_2_), as was also lacking for *in vitro* biofilms.

## Discussion

The recalcitrance of oral biofilm toward penetration of antimicrobials is known ever since Van Leeuwenhoek wrote in the 17^th^ century that “*the vinegar with which I washed my teeth killed only those animals which were on the outside of the scurf, but did not pass through the whole substance of it*”. Over recent years, the limited penetration of antimicrobials into a biofilm has been attributed to reduced solute diffusion in water, the presence of bacterial cells, EPS, abiotic particles or gas bubbles trapped in a biofilm [21]. Interestingly, whereas the influence of the chemistry and biology of biofilms on diffusion have been amply described and reviewed [21], [26], [27], antimicrobial penetration has never been related with quantifiable, physical properties of a biofilm. This study demonstrates for the first time since Van Leeuwenhoek his observation of the poor penetration of vinegar into an oral biofilm, that through a relaxation-structure-composition relation, biofilm properties can be derived that facilitate explanation of antimicrobial penetration into a biofilm on basis of quantitative biofilm properties. Incidentally, not only antimicrobials have difficulty penetrating a biofilm, but also nutrients may have difficulty penetrating a biofilm, causing reduced viability of organisms residing in deeper layers of biofilms [28].

The bacteria in a biofilm constitute the heaviest masses, and their re-arrangement during stress relaxation upon an induced deformation will thus be slow, which associates the relative importance of the slow Maxwell element with bacterial re-arrangement. Furthermore, the positive correlation between penetration and the importance of the slow Maxwell element confirms that organisms arranged in a more open, water-filled structure, allow easier penetration of antimicrobials. Different from the role of water-filled channels in diffusion [21], we found that water itself had a negative influence on the efficacy of antimicrobials during penetration. Since water has the smallest viscosity in a biofilm, the fast Maxwell element may be associated with the outflow of water through and its presence in biofilms. Consequently, dilution of antimicrobials after penetration into a biofilm to an ineffective concentration in deeper layers is evidenced by the negative correlation between the relative importance of the fastest Maxwell element and the penetration ratio. At this point, it must be emphasized that in our study chlorhexidine might have penetrated beyond the dead bands, as visible in Fig. S1, but clearly to a concentration insufficient to yield bacterial killing. Arguably, this raises the issue that penetration not only depends on possible physical difficulties of an antimicrobial in penetrating a biofilm, but moreover on the time allowed for penetration and antimicrobial concentration. In many clinical situations however, time and concentration cannot be increased at will. In the oral case highlighted here, the time most people allow themselves for an antimicrobial mouthrinse to be active in the oral cavity is 30 s utmost, while concentrations of chlorhexidine higher than 0.12 w% rapidly cause severe soft tissue damage and discolorations of teeth [29]. Equilibration of a biofilm with an antimicrobial as can be achieved *in vitro* is thus often impossible for the *in vivo* situation. Clearly, similar types of limitations with respect to time and/or concentration exist everywhere in the human body where antimicrobials are applied to combat biofilm-related infections, emphasizing the importance of good penetration in biofilm control through the use of antimicrobials.

The importance of a relaxation-structure-composition relation for biofilms and its role in understanding antimicrobial penetration was established both for *in vivo* grown biofilms as well as in two distinctly different model systems to grow biofilms *in vitro*. In the CDFF, there is a constant turn-over of bacterial growth, death and biofilm removal by the scraper blades [23] in addition to compaction by the blades. Whereas similar turn-over, death and removal by fluid flow can be expected in a PPFC, compaction is absent in a PPFC. In this respect, it is interesting that there was no difference in stress relaxation of biofilms formed by coccal or rod-shaped organisms in the CDFF, presumably because biofilms in the CDFF are mechanically compacted during formation (see Table 1). In the absence of mechanical compaction like in the PPFC, rod-shaped organisms have more difficulties in spontaneously forming a dense structure, as this requires organisms to take a favorable orientation with respect to one another. This becomes especially evident at the larger deformation induced of 50% and explains why biofilms formed by rod-shaped organisms in the PPFC had a different stress relaxation than coccal organisms, but not in the CDFF.

The two model systems to grow biofilms used in this study represent two extreme situations that may occur in the oral cavity. Highly compacted biofilms may be expected in fissures due to mastication, while compaction occurs less on interproximal biofilms. In addition, biofilm-left-behind in interproximal spaces inaccessible to contact-brushing will be in a more “fluffed-up” state [30], resembling biofilms grown in a PPFC. Indeed, biofilms grown in our intra-oral biofilm collection device, inaccessible to contact toothbrushing, are more fluffed up than in *in vitro* formed biofilms (compare Figs. S1E and F with Figs. S1A-D). Accordingly, stress relaxation characteristics after 10% and 20% deformation of biofilms formed in the PPFC more closely resemble those of *in vivo* formed biofilms than biofilms formed in the CDFF. This is especially so for 10% and 20% induced deformations, yielding information on the relaxation-structure-composition of the outermost surface of the biofilms, opposite to data derived upon inducing 50% deformation that invokes the deeper layers of the biofilms. This being true for the images selected, it must be realized that it is difficult if not impossible by human nature to obtain confocal laser scanning microscopic (CLSM) images of biofilms in an unbiased, observer-independent way. This is why conclusions on biofilm structure from quantitative, observer-independent stress relaxation analysis of larger sections of a biofilm than can ever be obtained microscopically, are to be preferred. Interestingly, upon increasing the induced deformation to 50%, a better resemblance between *in vivo* and CDFF grown biofilms appears. This is probably because biofilms formed *in vivo* are compacted more than when formed in a PPFC through the presence of multiple strains and species that can more easily arrange themselves spontaneously through their differences in size and shape to a compact mass, even in the absence of external compaction or mechanical perturbations. For single-species biofilms grown in a CDFF, this compaction is achieved by continuously scraping off the biofilm by a rotating blade. Therefore it can be expected that oral biofilm in fissures and interproximal spaces, left behind multiple times after brushing, will eventually become compacted and better resemble biofilms formed in the CDFF than oral biofilms freshly formed, for which the PPFC may be the preferred model system.

The *in vivo* relations between relaxation characteristics and chlorhexidine penetration have larger 95% confidence intervals than the in *vitro* ones, partly due to the limited power of the study that was confined to five volunteers. More importantly however, it is intrinsically impossible to obtain the same narrow confidence intervals for *in vivo* biofilms as found for *in vitro* biofilms, that were all single-species. In our analyses, we employ chlorhexidine killing as an indicator of its penetration. *In vivo* formed biofilms contain a large number of different strains and species, that all have their own susceptibility to chlorhexidine not only within one volunteer, but also among volunteers. This inevitably affects the penetration as indicated by bacterial killing of chlorhexidine, making the *in vivo* relation less significant than the one obtained for *in vitro* biofilms.

In summary, this study is the first to demonstrate a role of visco-elastic properties of oral biofilm on antimicrobial penetration through a relaxation-structure-composition relationship. Herewith, biofilm visco-elasticity becomes an important quantifiable physical property of biofilms next to qualitative, observer-dependent CLSM-imaging of structure, with respect to advancing our understanding of antimicrobial penetration in biofilms. Although the current study was performed on oral biofilms, its applicability will extend to biofilms formed in other industrial and biomedical applications. Especially in the biomedical field, understanding the factors that control the penetration of antibiotics into biofilms is of utmost importance, as difficult to treat biofilm-related infections occur across all medical sub-disciplines causing large patients morbidity and mortality and inflicting huge costs to the health care system.

## Materials and Methods

### Bacterial Strains and Growth Conditions

*S. oralis* J22 and *A. naeslundii* T14V-J1 grown on blood agar plates, were used to inoculate 10 ml modified Brain Heart Infusion broth (BHI, Oxoid Ltd., Basingstoke, UK) (37.0 g/l BHI, 5.0 g/l yeast extract, 0.4 g/l NaOH, 1.0 g/l hemin, 0.04 g/l vitamin K1, 0.5 g/l L-cysteine, pH 7.3) and were cultured for 24 h at 37°C in ambient air for *S. oralis* J22 and anaerobically for *A. naeslundii* T14V-J1. These cultures were used to inoculate 200 ml modified BHI and grown for 16 h. Bacteria were harvested by centrifugation at 870 g, 10°C for 5 min and washed twice in sterile adhesion buffer (50 mM potassium chloride, 2 mM potassium phosphate, 1 mM calcium chloride, pH 6.8). The bacterial pellet was suspended in 10 ml adhesion buffer and sonicated intermittently in an ice-water bath for 3 × 10 s at 30 W (Vibra cell model 375, Sonics and Materials Inc., Newtown, CT, USA) to break bacterial chains and clusters, after which bacteria were resuspended in adhesion buffer. A concentration of 3 × 10^8^ bacteria/ml was used for PPFC experiments, while a concentration of 9 × 10^8^ bacteria/ml was used in CDFF experiments.

### Biofilm Formation in a PPFC and CDFF

Biofilms were grown on glass slides (water contact angle 7±3 degrees) and hydroxyapatite discs (water contact angle 34±8 degrees) in a PPFC and a CDFF, respectively after adsorption of a salivary conditioning film from reconstituted human whole saliva for 14 h at 4°C under static conditions. Reconstituted human whole saliva was obtained from a stock of human whole saliva from at least 20 healthy volunteers of both genders, collected into ice-cooled beakers after stimulation by chewing Parafilm®, pooled, centrifuged, dialyzed, and lyophilized for storage. Prior to lyophilization, phenylmethylsulfonylfluoride was added to a final concentration of 1 mM as a protease inhibitor in order to reduce protein breakdown. Freeze-dried saliva was dissolved in adhesion buffer (1.5 g/l). All volunteers, gave their verbal informed consent to saliva donation according to a fixed written protocol and were registered in order to document the gender, age and health status of the volunteers, in agreement with the guidelines set out by the Medical Ethical Committee at the University Medical Center Groningen, Groningen, The Netherlands (approval letter 06-02-2009). Written consent was not required since saliva collection was entirely non-invasive, saliva’s were pooled prior to use and the study was not aimed towards measuring properties of the saliva. Rather saliva was used to lay down an adsorbed protein film prior to biofilm formation studies. For biofilm formation in the PPFC, 200 ml bacterial suspension was circulated at a shear rate of 15 s^−1^ in a sterilized PPFC till a bacterial surface coverage of 2×10^6^ cm^−2^ was achieved on a saliva-coated glass bottom plate (for details see (16)). Subsequently, adhesion buffer was flowed at the same shear rate of 15 s^−1^ for 30 min in order to remove non-adhering bacteria from the tubes and flow chamber. Next, growth medium (20% modified BHI and 80% adhesion buffer) was perfused through the system at 37°C for 48 h, also at a shear rate of 15 s^−1^.

Biofilms were grown in a sterile CDFF (for details see (23)) on saliva coated hydroxyapatite discs by introducing 200 ml bacterial suspension in the fermenter during 1 h, while the table with the sample holders was rotating at 1 rpm. Then, rotation was stopped for 30 min to allow bacteria to adhere before growth medium was introduced and rotation resumed. The biofilm was grown for 96 h at 37°C under continuous supply of a mixture of adhesion buffer and modified BHI at a rate of 80 ml/h. The system was equipped with 15 sample holders and each sample holder contained 5 saliva coated hydroxyapatite discs, recessed to a depth of 100 µm.

### Oral Biofilm Collection *in vivo*

The intra-oral biofilm collection device (Fig. S3) was made of medical grade stainless steel 316, and is composed of two parts: a base (5×3×2 mm) that is fixed to the center of the buccal surface of the upper first molars and a replaceable cover plate (4×3×0.2 mm). Biofilms formed on the inner side of the replaceable cover plate in the absence of mechanical perturbations, were considered for this study.

Five volunteers (aged 26 to 29 years) were included in this study. Volunteers all had a complete dentition with maximally one restoration, no bleeding upon probing and were not using any medication. Each volunteer was assigned a random number between 1 and 5 used for later data processing. The study was approved according to the guidelines of the Medical Ethics Committee of the University Medical Center Groningen, Groningen, The Netherlands (letter 28-9-2011), including the written informed consent by the volunteers and the tenets of the Declaration of Helsinki.

A base device was fixed to buccal surfaces of the upper first molars of the volunteers (see also Fig. S3) after mild etching of the tooth surface using light cure adhesive paste (Transbond™ XT, 3M Unitek, USA), a procedure similar to the one used for the bonding of orthodontic brackets. Prior to bonding, the base and cover plate of the device were brushed using a rubber cup and cleaner paste (Zircate® Prophy Paste, Densply, Caulk, USA) at low speed (less than 2,500 rpm/min) and autoclaved. Subsequently, the base surface was coated with a thin layer of primer and bonding agent (CLEARFIL SE BOND, Kurary Medical Inc., Japan). The stainless steel cover plate was inserted using a pair of tweezers and kept in place using Light Cure Adhesive Paste (Transbond™ XT, 3M Unitek, USA). Volunteers were asked to wear the device for a total of eight weeks during which they were requested to perform manual brushing with a standard fluoridated toothpaste (Prodent Softmint®, Sara Lee Household & Bodycare, Exton, USA) according to their habitual oral hygiene but to refrain from the use of an additional mouthrinse.

The cover plates could be removed with a dental explorer, after which cover plates with biofilm were placed in a moisturized petri dish for transport from the dental clinic to the laboratory. In a separate pilot study, it was established that two weeks of intra-oral biofilm formation in the device yielded biofilm thicknesses that were similar to the ones obtained *in vitro*. Therewith, *in vivo* biofilms could be collected four times from each volunteer. After each experiment, cover plates were sanded to remove biofilm and other residuals, prior to autoclaving.

After the experiments, the base of the device was removed from the tooth surface with a debracketing plier and residual adhesive was grinded off the tooth surface with a low speed hand piece. A base device was only used once in each volunteer. The tooth surface was polished and cleaned with rubber cup and cleaner paste. No signs of gingival inflammation were observed in any volunteer after removal of the base device.

### Low Load Compression Testing

The thickness and stress relaxation of the biofilms were measured with a low load compression tester, described before (16). Stress relaxation was monitored after inducing 10, 20, and 50% deformation of the biofilms within 1 s and held constant for 100 s, while monitoring the stress relaxation (see Fig. 1A). Each deformation was induced three times at different locations on the same biofilm.

Stress relaxation as a function of time was analyzed using a generalized Maxwell model containing three elements (see Fig. 1B) according to
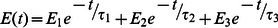
(1)in which E(t) is the total stress exerted by the biofilm divided by the strain imposed, expressed as the sum of three Maxwell elements with a spring constant E_i_, and characteristic decay time, τ_i_ (see also Fig. 1B). For calculating E(t), deformation was expressed in terms of strain, ε, according to the large strain model using
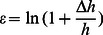
(2)where Δ*h* is the decrease in height and *h* is the un-deformed height of the biofilm. The model fitting for E_i_ and τ_i_ values of the three elements was done by minimizing the chi-squared value using the Solver tool in Microsoft Excel 2010. Fitting to three Maxwell elements yielded the lowest chi-squared values and increasing the number of Maxwell elements only yielded minor decreases in chi-squared values of less than 3%. The elements derived were rather arbitrarily named fast, intermediate or slow based on their τ values, i.e. τ_1_<5 s, 5 s<τ_2_<100 s and τ_3_>100 s, respectively (see also Fig. 1B). Relative importance of each element, based on the value of its spring constant E_i_, was expressed as the percentage of its spring constant to the sum of all elements’ spring constants at t = 0.

### Penetration of Chlorhexidine into Biofilms

*In vitro* and *in vivo* formed biofilms were all exposed *in vitro* to a 0.2 wt% chlorhexidine-containing mouthrinse (Corsodyl®, SmithKline Beecham Consumer Brands B.V., Rijswijk, The Netherlands) for 30 s and subsequently immersed in adhesion buffer for 5 min. After exposure to chlorhexidine, biofilms were stained for 30 min with live/dead stain (*Bac*Light™, Invitrogen, Breda, The Netherlands) and CLSM (Leica TCS-SP2, Leica Microsystems Heidelberg GmbH, Heidelberg, Germany) was used to record a stack of images of the biofilms with a 40× water objective lens. Images were analyzed with Leica confocal software to visualize live and dead bacteria in the biofilms. The ratio of the intensity of red (dead bacteria) to green (live bacteria), R/G, was plotted *versus* the biofilm thickness (see Fig. S1). The biofilm thickness where the ratio R/G became less than 1.5 was taken as the thickness of the dead band. Next, a penetration ratio was calculated according to
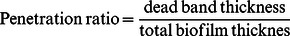
(3)


Penetration ratios were calculated for three different, randomly chosen locations on the biofilms and presented as averaged over the different locations.

### Statistical Analysis

Statistical analysis was performed with SigmaPlot software (version 11.0, systat software, Inc., California, USA). Differences in biofilm thickness and visco-elasticity were evaluated after testing for normal distribution and equal variance of the data. If data failed one of these tests, a Mann-Whitney Rank Sum test was used to determine statistical significance, otherwise a Student *t*-test was applied. Pearson Product Moment Correlation test was used to disclose relations between the penetration of chlorhexidine into and the relaxation of biofilms.

## Supporting Information

Figure S1 **Chlorhexidine penetration into** ***in vitro*** **and** ***in vivo*** **biofilms and calculation of the penetration ratio.** (I) Representative CLSM-images (cross sectional view) of the penetration of chlorhexidine (0.2 wt%) during 30 s into oral biofilms grown *in vitro* and *in vivo* (exposure to chlorhexidine was done *in vitro*). (A) *S. oralis* J22 biofilm grown under flow in a PPFC. (B) *S. oralis* J22 biofilm grown under compaction in a CDFF. (C) *A. naeslundii* T14V-J1 biofilm grown under flow in a PPFC. (D) *A. naeslundii* T14V-J1 biofilm grown under compaction in a CDFF. (E and F) two weeks old, *in vivo* formed oral biofilm. Scale bar represents 75 µm. (II) Red to green intensity ratio (R/G), denoting the ratio of dead to live organisms in a biofilm *versus* the thickness of the biofilm. *a* is the dead band thickness and *b* is the total biofilm thickness. R/G = 1.5 was taken as the cut-off for the thickness of the dead band.
(TIF)

Figure S2 **Tolerance and intolerance of biofilm organisms to chlorhexidine prior to and after their dispersal.** Fluorescence images of dispersed *S. oralis* J22 and *A. naeslundii* T14V-J1, treated with chlorhexidine for 30 s in their biofilm mode of growth prior to dispersal and treated immediately after dispersal. Live (green)–dead (red) staining was used to show the viability of bacteria. (A) *S. oralis* J22 grown in the PPFC and treated in its biofilm mode of growth. (B) *S. oralis* J22 grown in the PPFC and treated in its dispersed state. (C) *A. naeslundii* T14V-J1 grown in the CDFF and treated in its biofilm mode of growth. (D) *A. naeslundii* T14V-J1 grown in the CDFF and treated in its dispersed state. Scale bar represents 10 µm.
(TIF)

Figure S3 **Intra-oral biofilm collection device.** (A) The stainless steel base and cover plate of the device. (B) The base of the intra-oral biofilm collection device fixed to the center of the buccal surface of a maxillary first molar. (C) Side view of the intra-oral biofilm collection device, showing the open spacing in which undisturbed biofilm growth to the cover plate occurred. (D) Top view of the closed intra-oral biofilm collection device *in situ*, showing the hole in the cover plate used for its removal with a dental explorer.
(TIF)
