# Supplementary figures and images for: Stress Relaxation Analysis Facilitates a Quantitative Approach towards Antimicrobial Penetration into Biofilms
Source: PLoS One. 2013 May 27;8(5):e63750. doi: 10.1371/journal.pone.0063750 (PMC3664570; doi:10.1371/journal.pone.0063750)

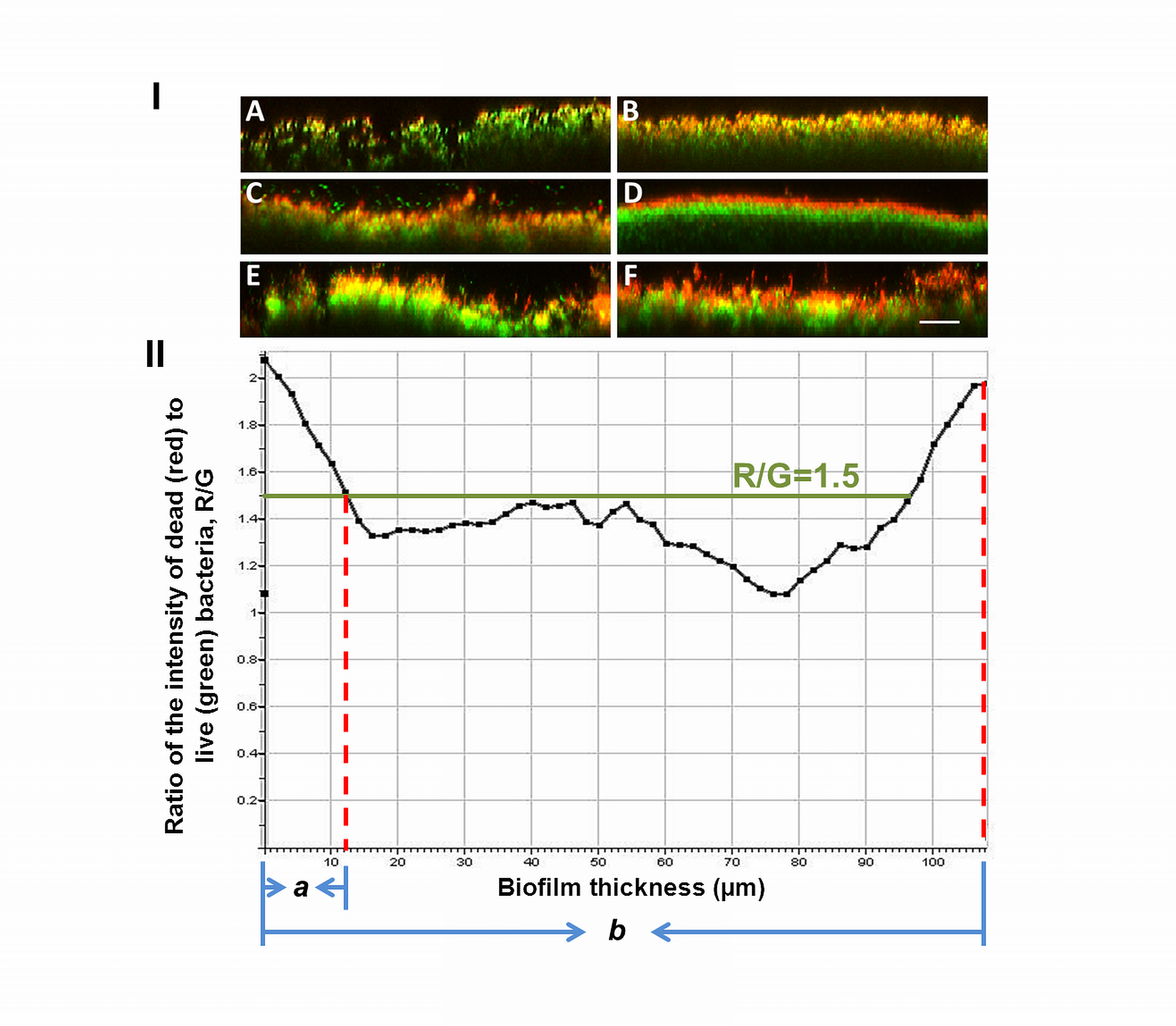

Supplement: Figure S1 — Chlorhexidine penetration into in vitro and in vivo biofilms and calculation of the penetration ratio. (I) Representative CLSM-images (cross sectional view) of the penetration of chlorhexidine (0.2 wt%) during 30 s into oral biofilms grown in vitro and in vivo (exposure to chlorhexidine was done in vitro). (A) S. oralis J22 biofilm grown under flow in a PPFC. (B) S. oralis J22 biofilm grown under compaction in a CDFF. (C) A. naeslundii T14V-J1 biofilm grown under flow in a PPFC. (D) A. naeslundii T14V-J1 biofilm grown under compaction in a CDFF. (E and F) two weeks old, in vivo formed oral biofilm. Scale bar represents 75 µm. (II) Red to green intensity ratio (R/G), denoting the ratio of dead to live organisms in a biofilm versus the thickness of the biofilm. a is the dead band thickness and b is the total biofilm thickness. R/G = 1.5 was taken as the cut-off for the thickness of the dead band. (TIF) [file pone.0063750.s001.tif]

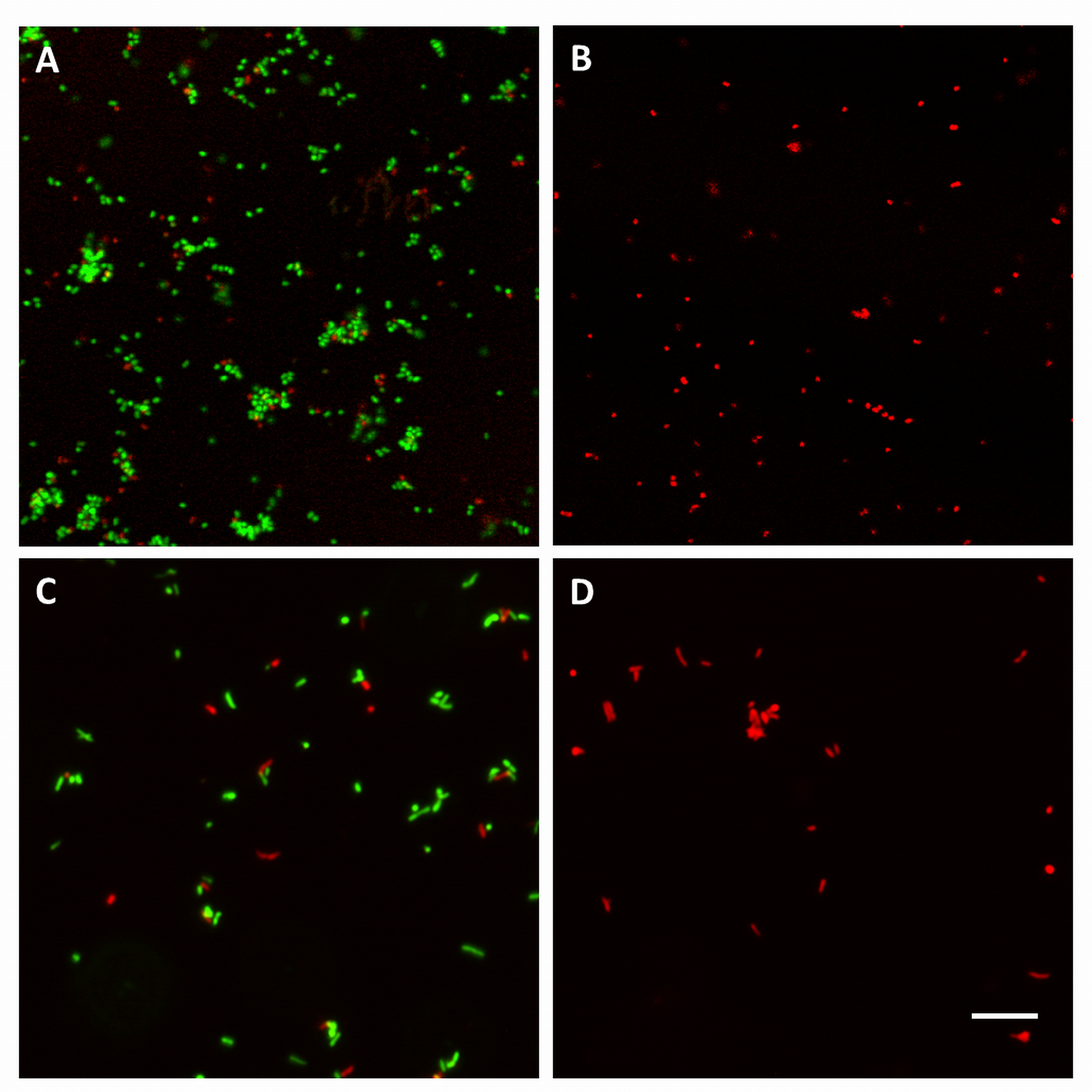

Supplement: Figure S2 — Tolerance and intolerance of biofilm organisms to chlorhexidine prior to and after their dispersal. Fluorescence images of dispersed S. oralis J22 and A. naeslundii T14V-J1, treated with chlorhexidine for 30 s in their biofilm mode of growth prior to dispersal and treated immediately after dispersal. Live (green)–dead (red) staining was used to show the viability of bacteria. (A) S. oralis J22 grown in the PPFC and treated in its biofilm mode of growth. (B) S. oralis J22 grown in the PPFC and treated in its dispersed state. (C) A. naeslundii T14V-J1 grown in the CDFF and treated in its biofilm mode of growth. (D) A. naeslundii T14V-J1 grown in the CDFF and treated in its dispersed state. Scale bar represents 10 µm. (TIF) [file pone.0063750.s002.tif]

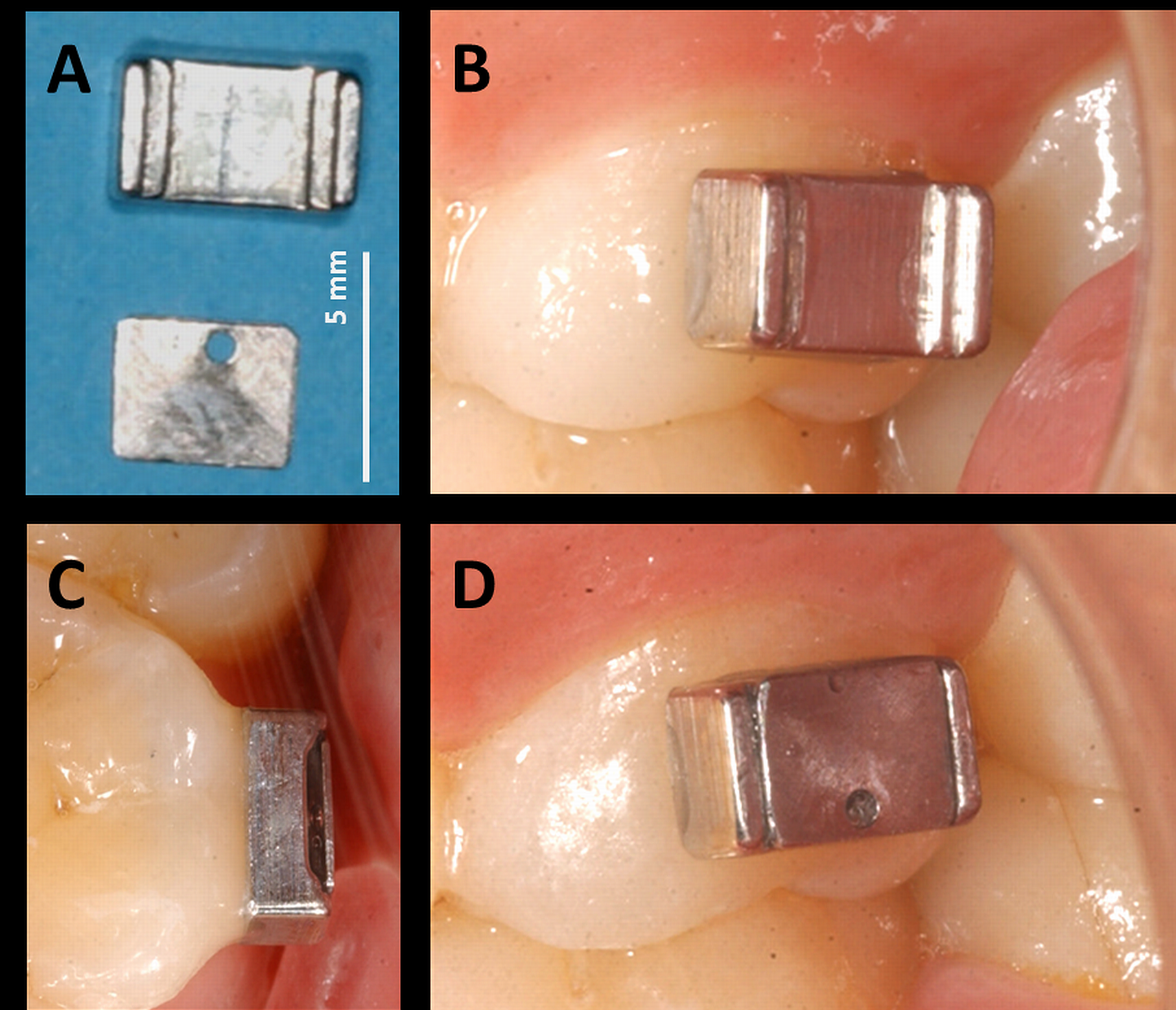

Supplement: Figure S3 — Intra-oral biofilm collection device. (A) The stainless steel base and cover plate of the device. (B) The base of the intra-oral biofilm collection device fixed to the center of the buccal surface of a maxillary first molar. (C) Side view of the intra-oral biofilm collection device, showing the open spacing in which undisturbed biofilm growth to the cover plate occurred. (D) Top view of the closed intra-oral biofilm collection device in situ, showing the hole in the cover plate used for its removal with a dental explorer. (TIF) [file pone.0063750.s003.tif]
